# Neuroticism, depression and anxiety in takotsubo cardiomyopathy

**DOI:** 10.1186/s12872-016-0277-4

**Published:** 2016-05-31

**Authors:** Thomas Emil Christensen, Lia E. Bang, Lene Holmvang, Philip Hasbak, Andreas Kjær, Per Bech, Søren Dinesen Østergaard

**Affiliations:** Department of Cardiology, Copenhagen University Hospital, Rigshospitalet, Copenhagen, Denmark; Department of Clinical Physiology, Nuclear Medicine & PET and Cluster for Molecular Imaging, Copenhagen University Hospital, Rigshospitalet and University of Copenhagen, Copenhagen, Denmark; Psychiatric Research Unit, Psychiatric Center North Zealand, Copenhagen University Hospital, Hillerød, Denmark; Department of Clinical Medicine, Aarhus University Hospital, Aarhus, Denmark; Department P - Research, Aarhus University Hospital, Risskov, Denmark

**Keywords:** Takotsubo cardiomyopathy, Well-being, Neuroticism, Depression, Anxiety

## Abstract

**Background:**

Takotsubo cardiomypathy (TTC) causes acute reversible heart failure. Prior studies have indicated that the syndrome is associated with traits such as social inhibition, chronic psychological stress, and anxio-depressive disorders. The objective of this study was to further characterize key psychological/psychopathological traits of patients with TTC.

**Methods:**

A survey of three groups was conducted: I) Female post-recovery TTC patients admitted between October 1^st^ 2009 and December 10^th^ 2014, II) Age, gender and geographically matched ST-elevation myocardial infarction (STEMI) patients, and III) Age, gender and geographically matched individuals from the background population. The following questionnaires were used in the survey: the WHO-5 Well-Being Index, Eysenck’s Neuroticism Scale, the Major Depression Inventory, and the anxiety subscale of Symptoms Checklist (SCL-90).

**Results:**

In total, 173 of 230 invitees (75 %) participated in the study. In comparison to the background controls, TTC patients reported significantly less well-being, more neuroticism, more depression, and more anxiety. The levels of well-being, depression and neuroticism were comparable between TTC and STEMI patients, but the level of anxiety was higher in the TTC patients. There was a negative correlation between the time since TTC admission and the total scores on the psychopathology rating scales.

**Conclusions:**

Patients with TTC reported significantly higher anxiety levels compared to both STEMI patients and background controls. However, unlike the STEMI patients, the TTC patients appeared to improve psychologically during the post-recovery phase. This may be a consolation for TTC patients in acute psychological distress.

## Background

Takotsubo cardiomypathy (TTC) is an increasingly recognized cause of acute heart failure mainly affecting elderly females [1]. The onset of TTC is often preceded by an emotional stressor [2], and catecholamine induced cardiomyopathy has been suggested as the underlying cause [3, 4]. TTC patients typically have elevated cardiac biomarkers, electrocardiographic abnormalities including ST-elevations, but absence of a coronary culprit lesion on angiography. Left ventricle contractility is abnormal with characteristic akinesia of the apical-midventricular segments, and basal normo- or hyperkinesia (‘apical ballooning’) [5]. Complete remission usually occurs within 1 month [6] and it was previously thought that TTC patients had an excellent prognosis. However, recent reports indicate a poor outcome similar to myocardial infarction [7].

It is well established that affective disorders constitute an independent risk factor for cardiovascular disease [8]. In the case of TTC, studies indicate that the syndrome is linked to psychopathological traits such as social inhibition, chronic psychological stress, and anxio-depressive disorders [9–12]. However, most studies have been conducted within a few months of initial admission, and thus recent life events related to the onset of TTC may have confounded the results. Therefore, the aim of this study was to characterize key psychological/psychopathological traits in a large, carefully diagnosed cohort of post-recovery TTC patients using well-validated psychometric tools. For comparison, we included one group of patients with previous ST elevation myocardial infarction (STEMI), and one group of background population controls. We hypothesized that specific psychopathological traits were more pronounced in the patients with TTC compared to the STEMI patients and the individuals in the background control group.

## Method

### Participants

A total of 45 TTC patients were prospectively recruited among female patients admitted at the Department of Cardiology, Copenhagen University Hospital from October 1^st^ 2009 to December 10^th^ 2014. Inclusion criteria were **(1)** acute onset of symptoms, **(2)** no culprit lesion on coronary angiography, **(3)** typical ‘apical ballooning’, **(4)** elevated cardiac biomarkers, and **(5)** normalized left ventricle systolic function on follow-up echocardiography. The STEMI patients were 95 age- and geographically matched females with previous one-vessel disease and STEMI that had received primary coronary intervention at Copenhagen University Hospital within the TTC inclusion period. The background controls were 90 age- and geographically matched females randomly selected from the Danish Civil Registration Registry [13].

### Survey

The survey was conducted between February and April 2015. A questionnaire for self-reporting was mailed to the invitees’ home addresses, and a maximum of two reminders were sent by letter if invitees did not respond to the first questionnaire. The questionnaire consisted of the following self-rating scales: The 5-item WHO-5 Well-Being Index (WHO-5) [14], the 23-item Eysenck’s Neuroticism Scale (ENS) [15, 16], the 10-item Major Depression Inventory (MDI) [17], and the 8-item anxiety subscale of Hopkin’s Symptoms Checklist (ASS) [18]. These self-rating scales are well-validated measures of well-being, neuroticism, depression, and anxiety, respectively. Not all questionnaires were fully filled out by the participants. If >20 % of the item scores on one of the four self-rating scales were missing, this entire scale was excluded from further analysis. If ≤20 % of the item scores were missing on a self-rating scale, the missing scores were replaced by the mean value of the completed item scores of this scale.

### Statistics

Unless stated otherwise, results are presented as median (interquartile range). The total score for each of the four rating scales was compared pair-wise between groups by means of the Wilcoxon-Mann-Whitney test. Correlations between rating scale total scores and age, and time since admission respectively, were tested by Spearman rank (r) correlation analysis. Both the Wilcoxon-Mann-Whitney test and the r correlation analyses were performed using SAS Statistical software® (version 9.0) with the level of significance set at two-tailed *p* < 0.05. As we intended to use the total scores of each of the four self-rating scales as measures for overall well-being (WHO-5) or syndrome severity (END, MDI, and ASS), we tested the scalability of these scales. Scalability is present when each of the individual item scores of a scale contributes unique information regarding the dimension of interest. Only when this is the case can the individual item scores be summed to a meaningful total score [19]. The analysis of scalability was performed using the Mokken non-parametric item response theory model, where the degree of scalability is expressed by the coefficient of homogeneity. A coefficient of 0.40 or higher indicates acceptable scalability [20]. The Mokken analysis was performed with the dedicated MSP 3.0 software [21].

### Ethics

The study was approved by the regional ethics committee, and all participants provided written consent.

## Results

**Fig. 1:**
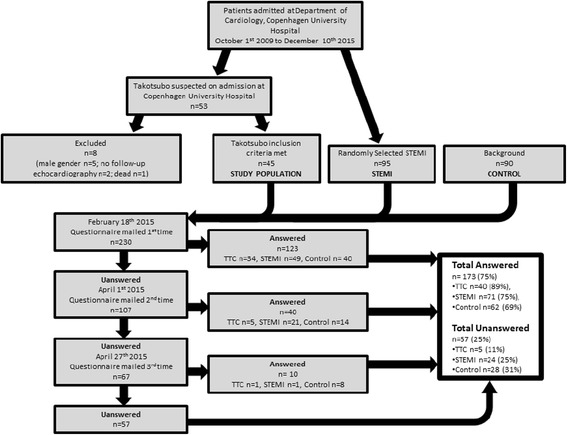
Flow chart depicting study inclusion

The flow of participants in the survey is depicted in Fig. 1. In total, 173 of 230 (75 %) invitees, 40 TTC patients (89 % of invitees), 71 STEMI patients (75 % of invitees) and 62 background controls (69 % of invitees), participated in the study. Age in years was 70 (64; 76) for the TTC patients, 72 (64; 77) for the STEMI patients, and 67 (64; 70) for the background controls. The age of the TTC and the STEMI patients was comparable (*p* = 0.79), whereas the background controls were significantly younger than both the TTC patients (p = 0.02), and the STEMI patients (*p* = 0.003). The time from initial admission to the survey was 24 (8; 36) months and 26 (22; 32) months for TTC and STEMI patients, respectively, with no statistically significant difference between the two groups (*p* = 0.70). The Mokken analysis showed that all of the self-rating scales included in the survey had acceptable coefficients of homogeneity (≥0.40): WHO-5 0.65, ENS 0.40, ASS 0.66, and MDI 0.53. This entails that the total scores of the scales are valid (scalable) measures of the psychological/psychopathological dimensions being investigated [19].

**Fig. 2:**
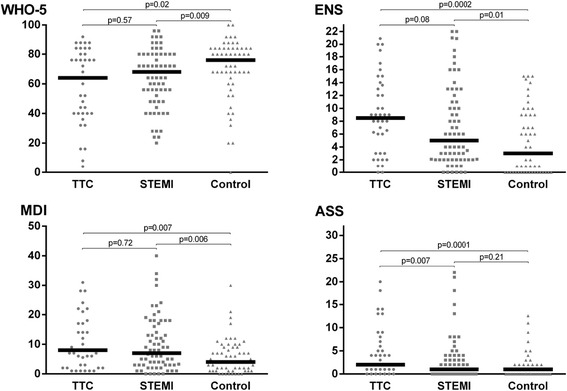
Scatterplots of total scores on the WHO-5, ENS, MDI and ASS for the three groups of participants. The bars represent the median and the p-values represent the results of the pair-wise Wilcoxon-Mann-Whitney tests of difference in total scores between groups

The results of the survey are presented in Fig. 2. Well-being and depression scores were comparable between the TTC and STEMI groups (WHO-5: 64 (40; 80) vs. 68 (52; 80), *p* = 0.57. MDI: 8 (3; 16) vs. 7 (3; 14), *p* = 0.72). ENS score tended to be higher in the TTC group (ENS: 9 (3; 13) vs. 5 (2; 11), *p* = 0.08), but the difference was not statistically significant. The anxiety score was significantly higher in the TTC than the STEMI group (ASS: 2 (1; 7) vs. 1 (0; 3), *p* = 0.007). When compared to the control group, TTC patients reported significantly less well-being (WHO-5: 76 (68; 84) vs. 64 (40; 80), *p* = 0.02), higher neuroticism (ENS: 3 (0; 9) vs. 9 (3; 13), *p* = 0.0002), more depression (MDI: 4 (1; 9) vs. 8.0 (3; 16), *p* = 0.007), and more anxiety (ASS: 1 (0; 2) vs. 2 (1; 7), *p* = 0.0001). Compared to the background controls, the STEMI patients reported less well-being (WHO-5: 76 [68; 84] vs. 68 [52; 80], *p* = 0.009), more neuroticism (ENS: 3 [0; 9] vs. 5[2; 11], *p* = 0.01), more depression (MDI: 4 [1; 9] vs. 7 [3; 14], *p* = 0.006), whereas the level of anxiety was similar (ASS: 1[0; 2] vs. 1 [0; 3], *p* = 0.21). There was no significant correlation between age and any of the total scores on the four self-rating scales (Age and WHO-5: *r* = 0.06, *p* = 0.46, *n* = 173; Age and ENS: *r* = 0.01, *p* = 0.94, *n* = 169; Age and MDI: *r* = 0.01, *p* = 0.94, *n* = 173; Age and ASS: *r* = −0.01, *p* = 0.93, *n* = 171).

**Fig. 3:**
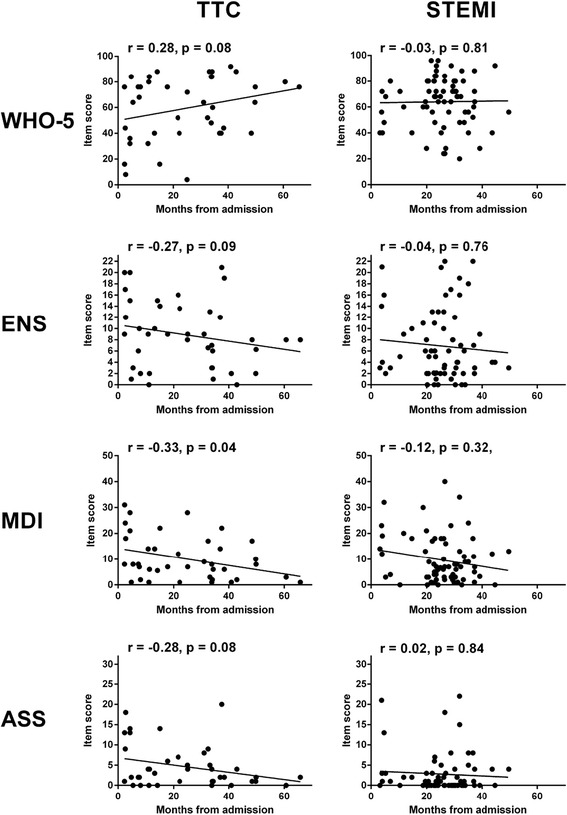
The time (in months) between admission and survey participation (*x-axis*) plotted against the total score (*y-axis*) on each of the four rating scales for the TTC (left) and STEMI (*right*) patients respectively. The fitted line of least squares is depicted. The Spearman rank coefficient (r) and the associated p-value are shown for each correlation

In Fig. 3, the time between admission and survey participation is plotted against the total scores of the WHO-5, ENS, MDI and ASS for the TTC and STEMI patients, respectively. For the STEMI patients, there was no significant correlation between time and total scores (Time and WHO-5: *r* = −0.03, *p* = 0.81, *n* = 70; Time and ENS: *r* = −0.04, *p* = 0.76, *n* = 69; Time and MDI: *r* = −0.12, *p* = 0.32, *n* = 70; Time and ASS: *r* = 0.02, *p* = 0.84, *n* = 68). Conversely, the same correlations were either significant or near-significant (*p* < 0.1) for the TTC patients (Time and WHO-5: *r* = 0.28, *p* = 0.08, *n* = 40; Time and ENS: *r* = −0.27, *p* = 0.09, *n* = 40; Time and MDI: *r* = −0.33, *p* = 0.04, *n* = 40; Time and ASS: *r* = −0.28, *p* = 0.08, *n* = 40), which is indicative of psychological improvement in the post-recovery phase of TTC.

## Discussion

We examined four psychological/psychopathological dimensions in post-recovery female TTC patients, age- and geographically matched female STEMI patients and age- and geographically matched females from the background population. As expected, the results showed that TTC patients reported significantly less well-being, more neuroticism, more depression, and more anxiety than the background controls. The level of well-being, depression and neuroticism in TTC patients was comparable to that of the STEMI patients, but the TTC patients were significantly more anxious than the STEMI patients. These findings are consistent with previous studies of the psychological/psychopathological profile of patients with TTC. Specifically, Compare et al. compared the prevalence of Type D personality in TTC patients with an emotional trigger to TTC patients without an emotional trigger and to STEMI patients. Type D personality is characterized by negative affect and social inhibition, and the frequency of Type D personality was higher in the TTC patients with an emotional trigger than in the two other groups [9]. Similarly, Delmas et al. showed that anxio-depressive disorders and chronic psychological stress was common in TTC patients and occurred more often than in patients with coronary disease [10]. Finally, Kastaun et al. found that TTC patients showed impaired cortisol release in response to stress, and were more anxious, worried and socially inhibited when compared to STEMI and healthy controls [11].

In contrast to STEMI, TTC leaves no permanent myocardial damage [22]. Thus, TTC patients are generally not considered to be chronically ill. Despite this fact, both the present study and the literature in general indicate that TTC is associated with more pronounced psychopathology compared to chronic ischemic heart disease. However, the results of our study also indicated that TTC patients, as opposed to STEMI patients, tend to improve psychologically during the post-recovery phase. To our knowledge, this is a novel finding, which may be a consolation for TTC patients in psychological distress in the acute phase.

Limitations to this study warrant a mention. First and foremost, since our survey was conducted post-TTC, we were not able to investigate the direction of causality of the psychopathology-TTC association, i.e., whether individuals develop TTC because they are psychologically vulnerable, or whether they develop psychopathology because of TTC? Since TTC is believed to be caused by sympathetic hyperactivity [3, 4] and patients with anxiety disorders show increased sympathetic reactivity in response to stress [23, 24], it seems plausible that the direction of causality goes from psychopathology to TTC. This hypothesis is supported by a small retro-perspective study by Summers et al. [12] in which past medical records of TTC patients were reviewed and compared to those of STEMI patients and background controls. Compared to the two other groups, the TTC patients had a much higher prevalence of anxiety disorders in their pre-cardiac-illness medical history. However, definitive prospective studies of this association are lacking from the literature. This is probably due to the fact that with the relatively low incidence of TTC (approximately 2 % of patients admitted on suspicion of acute coronary syndrome [1]), it will require extremely large surveys with very long follow-up to obtain pre-morbid psychological/psychopathological data on a sufficient number of TTC patients. Consistently, the small sample size is also a limitation of the present retrospective study.

Another limitation of our study is the risk of self-selection-bias as not all invitees completed the survey. Indeed, the age of the controls was slightly lower than that of the TTC and STEMI patients, but the Spearman rank correlation analysis revealed no significant correlation between age and rating scale total scores. It therefore seems unlikely that the lower age of the background controls has biased the results. Since many baseline data were not collected, we cannot exclude that confounders such as differences in educational or income level between groups have contributed to our results.

## Conclusion

In conclusion, we found that post-recovery TTC patients reported levels of well-being, neuroticism, and depression that are comparable to those of STEMI patients. However, TTC patients reported higher levels of anxiety than STEMI patients. In contrast to STEMI patients, the TTC patients appear to improve psychologically during the post-recovery phase. This may be a consolation for patients in psychological distress in the acute phase of TTC.

## Abbreviations

ASS, 8-item anxiety subscale of Hopkin’s Symptoms Checklist; ENS, 23-item Eysenck’s Neuroticism Scale; MDI, 10-item Major Depression Inventory; STEMI, ST-elevation myocardial infarction; TTC, Takotsubo cardiomyopathy; WHO-5, World Health Organization-5 Well-Being Index.
